# Age-related cognitive decline: Can neural stem cells help us?

**DOI:** 10.18632/aging.100446

**Published:** 2012-03-31

**Authors:** Benedetta Artegiani, Federico Calegari

**Affiliations:** DFG–Research Center and Cluster of Excellence for Regenerative Therapies Dresden, Medical Faculty, Technische Universität Dresden and Max Planck Institute of Molecular Cell Biology and Genetics, Dresden, Germany

**Keywords:** adult neurogenesis, neural stem cells, aging, regenerative therapies

## Abstract

Several studies suggest that an increase in adult neurogenesis has beneficial effects on emotional behavior and cognitive performance including learning and memory. The observation that aging has a negative effect on the proliferation of neural stem cells has prompted several laboratories to investigate new systems to artificially increase neurogenesis in senescent animals as a means to compensate for age-related cognitive decline. In this review we will discuss the systemic, cellular, and molecular changes induced by aging and affecting the neurogenic niche at the level of neural stem cell proliferation, their fate change, neuronal survival, and subsequent integration in the neuronal circuitry. Particular attention will be given to those manipulations that increase neurogenesis in the aged brain as a potential avenue towards therapy.

## INTRODUCTION

Stem cells are characterized by their ability to divide to generate additional stem cells and differentiated cell types. Proliferative division can be symmetric or asymmetric, thereby one stem cell generates either two daughter stem cells or one stem cell plus one differentiated cell type, thus, triggering the expansion or self-renewal of the stem cell pool, respectively. Due to these unique features, stem cells are essential during embryonic development for tissue formation and during adulthood to ensure tissue homeostasis, repair, and regeneration [1-5].

The balance between cell loss and cell replacement is brilliantly maintained in most tissues by resident stem cells up to a point when such equilibrium starts to be progressively lost and newborn cells are not able to compensate for the ones that died, resulting in decline in tissue integrity and function and a diminished capacity of regeneration upon damage [2, 6]. The physiological loss of tissue homeostasis during life is typically concomitant to a progressive and extensive decline in the physical and cognitive performance of the whole organism that is commonly referred to as aging. In addition to loss of stem cell turnover, aging is also caused by a decrease in the overall function of differentiated cells that is due to a number of cell-intrinsic and environmental factors including DNA damage, reduction in telomere length, oxidative stress, induction of stress response pathways, and production and accumulation of misfolded proteins, which may result in a number of disorders including cancer, heart failure, neurological diseases, and many others [7-10].

For humans, aging is one of the most evident biological processes whose molecular and cellular mechanisms are still poorly understood. Since recent studies in the field of stem cell biology have brought new light into the regenerative potential of this cell population, several laboratories have been prompted to investigate the use of stem cells for attenuating the effects of aging. It has been suggested that exhaustion of stem cells may be a primary cause of aging [11, 12] and, in fact, forcing regeneration leads to stem cell exhaustion and premature aging [13]. Certainly, stem cells can contribute to aging in different tissues as a reflection of their relative contribution to cell turnover and regeneration implying that organs with a low turnover should be minimally affected by aging of stem cells [3]. However, while comparing different species, the lack of an obvious correlation between the abundance of stem cells, regenerative capacity, aging, and lifespan hardly allows to make any link [3]. Moreover, recent evidences indicate that the relative abundance of stem cells in certain organs does not necessarily correlate with their impact on organ function. Specifically, the mammalian brain is perhaps the organ with the lowest regenerative potential but the one in which the signs of aging are more manifested. Using the words of the renaissance writer Michel de Montaigne, “*age imprints more wrinkles on the mind than it does on the face*” indicating that age-related cognitive decline has the highest impact on the quality of life. To which extent this decline is dependent on neural stem and progenitor cells (together referred to as NSCs) is hard to tell but growing evidences indicate that, despite their negligible numbers, the few resident NSCs that are located in specific brain regions, most notably the subgranular zone of the hippocampus, seem to play a major role in cognitive functions such as learning, memory, and emotional behavior by generating, through intermediate progenitors, neurons that are constantly added to the brain circuitry throughout life [14-18].

Perhaps not coincidentally, aging constitutes one of the major factors reducing the proliferation of NSCs [19-21] while cognitive decline, including a reduced learning and memory performance, is commonly observed in aged individuals [22-24]. Certainly, NSCs are not the only cause of brain aging because non-neurogenic areas, most prominently the prefrontal cortex, are known to be responsible for many phenotypes connected to senescence including forgetfulness and distractibility [25-27]. A positive correlation between NSC activity, hippocampal function, and cognitive performance during aging has been proposed for groups of animals and single individuals [28-31] but other studies have challenged this view [32-35]. Nevertheless, manipulations that decrease neurogenesis typically worsen cognitive performance in senescent animals while, conversely, an increase in neurogenesis tends to improve learning and memory [29, 36-38]. These findings in basic stem cell research during aging have led to the hope that stem cell-based approaches might be useful to compensate, at least in part, the age-related cognitive decline that is occurring in human. In this context, it is important to notice that the final amount of newborn neurons integrating in the brain circuitry during life is controlled at different levels including NSCs proliferation, cell fate change, neuronal survival, and maturation. Given the importance of understanding the biology of NSCs during aging, in this review we discuss the present knowledge about the age-related changes in neurogenesis and its underlying mechanisms at the systemic, cellular, and molecular level. Particular attention is given to the manipulations that allowed the increase of neurogenesis as a potential means to improve cognitive performance during aging.

### NSCs proliferation and fate

**Figure 1:**
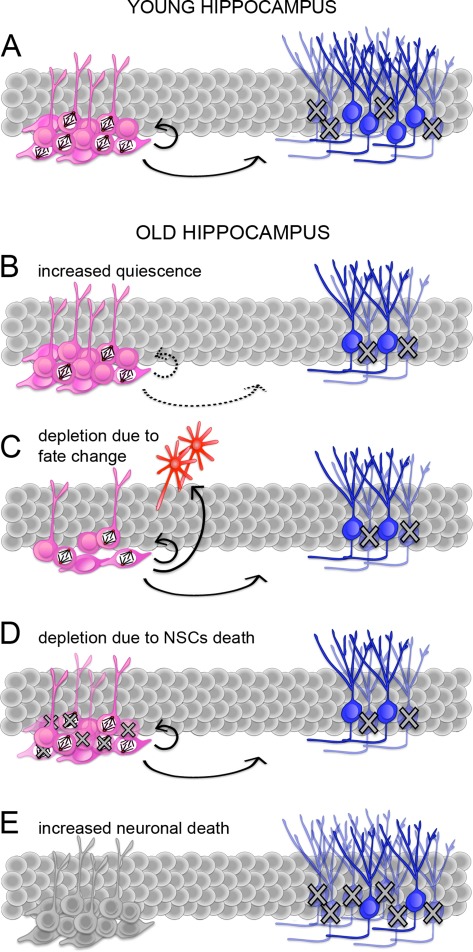
Potential mechanisms responsible for the age-related decline in neurogenesis (**A-E**) Schematic representation of adult neurogenesis in the young (**A**) or senescent (**B**-**E**) hippocampus. Proliferating and quiescent neural stem and progenitors cells (NSCs; pink) are indicated by the presence or lack of mitotic spindles, respectively. Proliferating NSCs divide to generate (arrows) additional NSCs or immature neurons (blue). During maturation, many neurons undergo apoptosis (crosses). A reduced number of neurons in the senescent hippocampus (**B**-**E**) can be explained by an increase in quiescence i.e. a lower proportion of proliferating NSCs (**B**), a change in NSCs fate i.e. an increase in differentiative at the expense of proliferative and/or gliogenic at the expense of neurogenic division (**C**), depletion of NSCs by cell death (**D**), or a higher proportion of newborn neurons undergoing apoptosis (**E**). Continuous or dashed arrows (**A**-**D**) indicate a constant or decreases proportion of proliferating NSCs and neurons generated from the total pool of NSCs, respectively. Note that NSCs in E are not colored because neuronal death could potentially occur concomitantly to any of the previous conditions (**A**-**D**).

Since the original observation of Altman and Das [19], several studies have consistently confirmed a major reduction in the abundance of proliferating NSCs during aging from rodents to primates [20, 21, 39]. In most studies, BrdU labeling, eventually in combination with markers of cycling cells, e.g.: Ki67 or PCNA, was commonly used to quantify the amount of cells undergoing cell cycle progression. Probably as a result of decreased proliferation, aging was shown to correlate with a decrease in newly generated neurons [20, 21, 39].Regarding NSCs, an important question remains as to whether the decrease in the number of proliferating cells reflects an increase in quiescence as opposed to their depletion. Moreover, an increase in the proportion of quiescent NSCs could be due to a higher number of cells leaving the cell cycle to become quiescent and/or a lower number of quiescent cells entering the cell cycle. Similarly, depletion of NSCs may be due to an increase in differentiation with concomitant loss of self-renewal and/or increased cell death (Figure 1). Discriminating between these possibilities becomes particularly important for studies aimed at compensating the age-related decline in neurogenesis though manipulations of endogenous NSCs either by controlling their cell cycle entry versus exit or, alternatively, by manipulating their cell fate change versus survival.

Certain studies based on the expression of NSCs markers that are independent from their proliferative state indicated a strong reduction in cell numbers in the aging hippocampus of rodents [40-42]. Conversely, other studies reported a change in their proliferative state but not in their absolute numbers [43, 44]. These discrepancies can partly be explained by considering that diverse strains, transgenic models, or even species were used and, in fact, it has been reported that senescent rodents and primates are characterized by a constant or decreased number of NSC, respectively [45]. In addition, it should be considered that the different markers used to characterize stem and progenitor cell types in different studies might identify slightly different cell populations. Assuming that the effects of aging may vary among progenitor subtypes, the discrepancies observed could, thus, reflect a diverse proportion of cell subpopulations identified using any specific marker within a given species.

The dichotomy between depletion of NSCs versus their entry in quiescence during life also emerged in two recent works, each supporting one of two alternative scenarios. According to the disposable stem cell model [46], quiescent NSCs enter the cell cycle and undergo a limited number of asymmetric cell divisions after which they terminally differentiate into mature astrocytes, thus, leading to depletion of the stem cell pool during life. Clonal analyses by a second group reported a diverse behavior of NSCs with a component of depletion but, in addition, a significant proportion of quiescent as well as cycling NSCs being detected over long periods of time [47].

The contradicting results concerning depletion versus quiescence of NSCs during aging are still debated and even more confusion is caused by failing to appreciate the differences between a reversible cell cycle arrest and a permanent loss of proliferative potential, in which only the former can properly be defined to as cellular senescence [48]. Nevertheless, a number of studies have consistently shown that proliferation of NSCs in old animals can be induced under certain conditions, including physical activity [38, 49-51] and disease [44, 52-55] indicating that, despite a partial depletion and, possibly, cellular senescence, a pool of NSCs is maintained that can be efficiently induced to re-enter the cell cycle and potentially contribute to improvement of brain function.

In addition to proliferation, NSCs activity can also be regulated at the level of a change in their fate (e.g.: by changing the proportion of cells undergoing proliferative versus differentiative, symmetric versus asymmetric, and/or gliogenic versus neurogenic divisions). Unfortunately, “decision making” in term of cell fate change as a factor accounting for the reduction in neurogenesis during aging is also debated. In fact, a decrease in the number of cells acquiring a neuronal phenotype has been proposed [36, 38] but other studies reported that even if the number of proliferating NSCs and newborn neurons decreases during aging, the proportion of differentiated cell types generated from the pool of cycling NSCs is similar or only slightly changed [35, 56-58].

Nevertheless, it should be considered that developing approaches to compensate for the age-related decline in NSC activity could be achieved at any level, including those that are not necessarily changed physiologically. Thus, it becomes important not only to identify the physiological alterations occurring in the NSC niche but also to consider the systemic, cellular, and molecular manipulations that could be used to control this process.

### Systemic effects of aging on NSCs

Among the systemic changes influencing NSCs activity, age-related alteration in the vasculature seems to assume a critical role. The tight relationship and relative dependency between adult neurogenesis and the vascular niche has been consistently described [59-62]. However, it is not without a certain sense of frustration that one realizes that, similarly to NSCs quiescence, depletion, and fate, the effects of aging on the brain vasculature are also highly controversial. While no change in the volume of capillaries in the dentate gyrus has been reported [38], the opposite result has also been observed with a decrease in capillary volume and proportion of NSCs clustering in their proximity [43] and even without considering specifically the neurogenic niches conflicting reports have been alternatively published [63].

Perhaps more conclusively, aging was shown to correlate with changes in hemorheological parameters resulting in a diminished cerebral blood flow and a consequent reduced availability of oxygen, metabolites, and neurotrophic factors [64-66] that are important for NSCs and whose alterations were associated with impairment in spatial memory [67].

Availability of oxygen may constitute an important factor responsible for the reduced neurogenesis of the aged hippocampus. As it had emerged from a number of in vitro studies using embryonic-derived NSCs, reducing the atmospheric oxygen tension to physiological levels i.e. from ca. 20% to 3%, increases NSCs proliferation [68-71]. A similar effect is now being revealed in the adult brain by studies linking the activity of hypoxia-inducible transcription factors (HIF) to the induction of genes known to be involved in stem cell differentiation including Wnt, Notch, and BMP [72-75]. Interestingly, aging mice display impaired hypoxic response including a deficiency in the activation of HIF-1 downstream targets [76] and an initial delay in the angiogenic response in hypoxic conditions [77].

In addition to oxygen, systemically released factors have a prominent role in NSCs activity. A recent study showed that during heterochronic parabiosis the old mouse displays higher level of NSCs proliferation and higher number of newborn neurons due to a change in soluble factors, including chemokines, present in the plasma of the young animal [78] but no molecular link between these chemoattractants and NSCs proliferation has been established. Other blood-released factors with a role on adult neurogenesis and aging deserving a special mention include steroid hormones and growth factors such as corticosterone (CORT), insulin-like growth factor-1 (IGF-1), fibroblast growth factor-2 (FGF-2), and vascular endothelial growth factor (VEGF).

Glucocorticoids are steroid hormones involved in age-related changes in the hippocampus, whose production and secretion by the adrenal gland are regulated by the hypothalamic-pituitary-adrenal axis, mainly in response to stress [79]. The mineralcorticoid and glucocorticoid receptors, strongly expressed in granule cells of the hippocampus [80, 81], and the latter also in NSCs [82], regulate gene expression upon activation by DNA binding of the glucocorticoid response element [79]. CORT is the primary glucocorticoid in rodents that downregulates cell proliferation in the hippocampus, as shown by CORT administration or adrenalectomy experiments in both young and senescent animals [42, 83-86] in which a decrease in CORT levels was shown to correlate with improved learning and memory [29]. In old rodents, CORT basal level is enhanced but stress-induced CORT increases only in the blood stream but not in the hippocampus [87]. In addition, expression of CORT receptors in NSCs and immature neurons of the hippocampus increases during aging[82].

Contrary to CORT, the three growth factors IGF-1, FGF-2 and VEGF were shown to have very similar effects in increasing NSCs proliferation. Expression and/or concentration of these factors and their receptors is known to decrease in the hippocampus during aging [88-91] and, in particular, levels of IGF-1 were shown to be higher, and cognitive performance to be improved, in long-living mutants [92]. A substantial number of experiments were performed to study the effects of the three factors on neurogenesis during adulthood or aging indicating a strong positive effect on NSCs proliferation by IGF-1 [93-95], FGF-2 [96-100], and VEGF [101-104] signaling, with the latter also resulting in improved spatial memory functions [104]. To which extent these or other factors released in the blood can directly influence cognitive function during aging is currently debated [105, 106].

The previous findings describing the role of the vasculature, oxygen tension, and blood-released factors deserve a special emphasis in the context of the physiological stimulus known to have the strongest positive effect on NSCs proliferation: physical exercise. Physical exercise, like voluntary running, is a physiological activity known to enhance NSCs proliferation and neurogenesis in the adult hippocampus and, concomitantly, improve cognitive functions in both young [107, 108] and old [38, 49-51] animals. The increase in cell proliferation induced by running was shown to depend on the release of IGF-1 [109, 110] and on the complex VEGF-dependent effects induced at the level of the vasculature [111].

Another physiological stimulus triggering systemic changes that correlate with a decrease in stem cell exhaustion and cellular senescence while, conversely, increasing lifespan is dietary restriction [112, 113]. Effects of dietary restriction are in part mediated by an inhibition of the mammalian target of rapamycin (mTOR) leading to a decreased senescence of stem and progenitor cells [48, 113-115] and several evidences indicate that these observations can be extended also to NSC of the adult brain [116-120].

Paradoxically, the two strongest physiological stimuli known to date to increase adult neurogenesis and lifespan, physical exercise [38, 49-51]and dietary restriction [112], respectively, are the ones that current societies of the western world more consistently neglect while asking biomedical research to fill the gap. To achieve this, manipulation of the factors released by the vasculature may provide a means to better understand and intervene in the patho-physiology of the aging brain [23, 121, 122].

### Cellular and molecular effects of aging on NSCs

**Figure 2:**
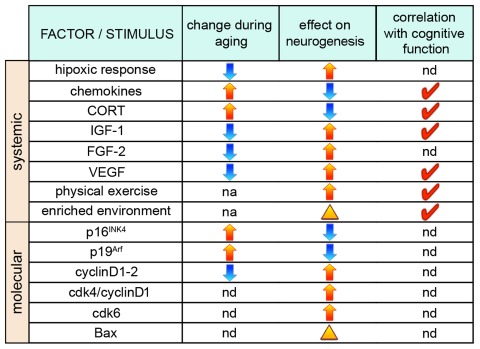
Factors influencing neurogenesis during aging (From left to right) Factors and stimuli influencing neurogenesis, their physiological increase (yellow arrows) or decrease (blue arrows) during aging, as well as effects on neurogenesis through, primarily, a change in NSCs proliferation (arrows) or neuronal survival (arrowhead) are indicated. Red ticks indicate factors whose manipulation has been shown to correlate with cognitive function. nd=not determined; na=not applicable.

In addition to systemic effects, NSCs proliferation can also be influenced by cellular and molecular changes taking place within the neurogenic niche (Figure 2). In fact, not only some of the factors mentioned above but also a long list of other signaling molecules and transcription factors known to regulate neurogenesis are produced locally by NSCs, astrocytes, or endothelial cells such as Notch, Wnt, BMP, Shh and several others [123-125]. Unfortunately, in the aging brain most of these factors were not investigated but it is interesting to notice that most, if not all, have been shown to inhibit differentiation and, in addition, shorten the cell cycle of NSCs [126-129]. Since the age-related reduction in NSCs proliferation is known to correlate with reduced levels of growth factors [89, 90], it would not come as a surprise if expression of genes regulating cell cycle length (or quiescence) should also be altered in the aging brain. In fact, expression of the inhibitors of G1 progression p16^INK4^ and p19^Arf^, that were previously linked to cellular senescence [130], were found to increase in the subventricular zone of old animals [131, 132] and deletion of p16^INK4^ was shown to promote NSC proliferation and neurogenesis in the aged subventricular zone but, apparently, not in the hippocampus [131]. Conversely, positive regulators of the cell cycle, most notably cyclinD1, were found to decrease during aging [133]. While not considering the differences that could exist within progenitors subtypes [40], measurements of cell cycle parameters in the dentate gyrus of young and 10 months old (middle-aged) rats did not detect changes in cell cycle length but only in the proportion of cycling cells [134] and similar measurements in the subventricular zone of 18 months old (senescent) mice corroborated this conclusion [135]. Interestingly, growing aged NSCs in culture was reported to shorten their cell cycle and induce proliferative, as opposed to neurogenic, divisions [135].

In addition to the cell fate determinants and growth factors mentioned above, also cell cycle regulators can control the fate of NSCs during embryonic development and adulthood [126-129] and even if not physiologically responsible for the aging phenotype, manipulation of cell cycle length could still be used as a strategy to overcome the reduction in neurogenesis occurring in aged animals. An impressive number of studies on cell cycle regulators and neurogenesis were already thoroughly reviewed [126-129]. Focusing exclusively on acute manipulations that increased neurogenesis, our group has recently found that expansion of NSCs can be achieved during embryonic development [136] and adulthood[137] through overexpression of the positive regulators of G1 progression cdk4/cyclinD1. This finding is consistent with the opposite effect reported in the adult brain after deletion of similar G1 regulators such as cdk6 [138] and cyclinD2 [139] and corroborate the notion (*the cell cycle length hypothesis*[140]) that manipulation of G1 length may influence the fate of somatic stem cells [128, 141]. Additional studies are needed to extend *the cell cycle length hypothesis* from the adult [137-139] to the senescent brain but findings using aged NSCs in vitro [135] makes this possibility likely.

### Neuronal maturation and survival

In addition to cell proliferation and fate, neuronal survival strongly influences the number of neurons produced in the adult hippocampus. It is known that the vast majority of newborn neurons are eliminated by apoptosis in the following 6-8 weeks and that only a small fraction is selected for long-term survival [142]. However, neuronal survival does not seem to be altered in aged animals since birthdating experiments based on the comparison of BrdU positive neurons at different times upon labeling in old and young animals showed that the proportion of neurons dying after birth is unchanged [56-58]. Migration, dendritogenesis, and expression of mature markers of newborn neurons were delayed in old animals [56] but this effect was compensated at later stages [56, 143].

Even if aging does not influence neuronal death, physiological stimuli can still be used to increase survival in senescent animals. In particular, living in an enriched environment that provides access to social and inanimate stimuli, such as toys, proved to be the strongest physiological condition increasing the survival of neurons in young and aged mice [36, 144] suggesting that the aged brain retains a certain level of neuroplasticity. Similar results were obtained after long-term exposure to enriched environment indicating an overall increase in the neuronal survival baseline rather than an acute response to new stimuli [37]. Importantly, increased neuronal survival in old animals positively correlated with a better performance in spatial memory tests [36, 37] end even if the molecular mechanism underlying this correlation is largely unknown, recent studies suggest a role of steroid hormone receptors [82].

In young mice, neuronal survival and, consequently, integration can be genetically enhanced, as recently shown after ablation of the proapoptotic gene Bax, leading to improvement in certain cognitive functions such as pattern separation [145]. As proposed by the authors of this work, promoting neuronal survival could constitute a new way to increase the number of neurons in old animals and, possibly, compensate age-related learning and memory deficits.

### Concluding remarks

A substantial number of evidences indicate that age-related decrease in adult neurogenesis is an important factor influencing cognitive performance. While several mechanisms may influence the number of mature neurons functionally integrated into the brain circuitry over time, the available data strongly suggests that aging almost exclusively acts at the level of NSC proliferation. Yet, the many contradicting results and uncertainties on identifying the exact causes of this “decreased proliferation” (i.e. quiescence, cellular senescence, cell cycle lengthening, and/or depletion via cell death or fate change) need to be fully acknowledged in order to give a rigorous and meaningful direction to this relatively new field. Nevertheless, in the context of therapy, also NSC fate, neuronal survival, and integration could potentially become the focus of interventions aimed at compensating for the decline in neurogenesis occurring during aging.

In this perspective, significant resources are invested in stem cell research in the hope that basic knowledge could one day be used for developing treatments of age-related cognitive decline and therapy of neurodegenerative diseases [146-148]. In this frame, it is interesting to notice that regulation of NSCs proliferation, although not necessarily associated with an increase in the number of integrated neurons, constitute a physiological response to certain diseases and that this response is maintained in aged rodents upon seizure [44, 52] and ischemia [53-55]. At least for stroke, a proliferative response has been observed in non-neurogenic areas also in human [149-151].

The fact that NSCs can efficiently respond to physiological and pathological stimuli to increase neurogenesis indicates that stimulation of endogenous NSCs offers a promising alternative to transplantation approaches that until now were intensely investigated but with very limited success [146-148, 152]. Proof of principle that increased neurogenesis in the adult hippocampus by acute manipulation of NSCs proliferation and fate [137] or neuronal survival [145] has been provided. Certainly, these reports fell short from demonstrating that a similar strategy can be applied in senescent mice to improve cognitive performance. Moreover, the studies discussed here indicate that a diminished neurogenesis during aging is primarily due to an increased quiescence and/or depletion of NSCs and since cdk4/cyclinD1 apparently changes the fate of NSCs but not their quiescence [137], while Bax knock-out inhibits neuronal death without increasing NSCs proliferation [145], it becomes unlikely that either of the two approaches would alone be sufficient to significantly improve cognitive function in senescent mice.

The strong positive correlation between increased endogenous neurogenesis and improved cognitive performance in senescent mice has already been provided upon life-long manipulation of blood-released factors [29] or acute exposure to physiological stimuli [38, 49-51]. It remains to be shown whether these effects can be reproduced by more specific manipulations triggering the appropriate combination of changes at the level of NSCs quiescence, fate, neuronal survival, and/or integration to improve cognitive performance in senescent individuals; even despite a lack of dietary moderation and physical exercise.
